# Performance of Elecsys^®^ HCV Duo Immunoassay for Diagnosis and Assessment of Treatment Response in HCV Patients with or without HIV Infection

**DOI:** 10.3390/diagnostics14192179

**Published:** 2024-09-29

**Authors:** Prooksa Ananchuensook, Jongkonnee Wongpiyabovorn, Anchalee Avihingsanon, Pisit Tangkijvanich

**Affiliations:** 1 Division of Gastroenterology, Department of Medicine, Faculty of Medicine, Chulalongkorn University, Bangkok 10330, Thailand; prooksa.anan@gmail.com; 2 Academic Affair, Faculty of Medicine, Chulalongkorn University, Bangkok 10330, Thailand; 3 Department of Microbiology, Faculty of Medicine, Chulalongkorn University, Bangkok 10330, Thailand; jongkonneew@gmail.com; 4 The HIV Netherlands Australia Thailand Research Collaboration (HIV-NAT), Bangkok 10330, Thailand; anchalee.a@hivnat.org; 5 Center of Excellence in Hepatitis and Liver Cancer, Department of Biochemistry, Chulalongkorn University, Bangkok 10330, Thailand

**Keywords:** Elecsys^®^ HCV Duo immunoassay, chronic hepatitis C virus (HCV) infection, direct-acting antivirals (DAAs), sustained, virological response (SVR), HCV core antigen, HCV antibodies

## Abstract

Background/Objectives: The Elecsys^®^ HCV Duo immunoassay (Roche Diagnostics International Ltd., Rotkreuz, Switzerland) detects both antibodies to hepatitis C virus (anti-HCV) and HCV core antigen (HCV-Ag) and has shown excellent diagnostic performance in blood donor samples. We aim to validate its use for diagnosing chronic HCV infection and assessing sustained virological response (SVR) post-direct-acting antivirals (DAAs) in patients with or without HIV infection. Methods: Blood samples from 100 healthy controls, as well as 64 HCV mono-infection and 136 HCV-HIV coinfections, were collected before and 12–24 weeks after DAAs. The assay performance for determining active infection at baseline and SVR was compared with HCV RNA. Results: Overall, 156 (78.0%) of HCV-infected patients had HCV genotype 1, and the SVR rate was 96.5%. The sensitivity, specificity, and area under the ROC curve (AUROC) for HCV diagnosis at baseline were 99.50% (95% confidence interval [CI], 96.82–99.97%), 100% (95%CI, 95.39–100%), and 0.998 (95%CI, 0.992–1.003), respectively. The corresponding results for HCV-Ag in determining SVR were 57.14% (95%CI, 20.24–88.19%), 97.41% (95%CI, 93.73–99.04%), and 0.773 (95%CI, 0.543–1.003), respectively. The assay also exhibited comparable sensitivity and specificity between HCV mono- and coinfection. Conclusions: Our study showed that the Elecsys^®^ HCV Duo immunoassay effectively diagnosed HCV infection, regardless of HIV status, making it suitable for managing high-risk populations in resource-limited settings.

## 1. Introduction

Hepatitis C viral (HCV) infection is one of the leading causes of liver-related complications, including cirrhosis and hepatocellular carcinoma (HCC) [1]. Based on its natural history without antiviral treatment, it is estimated that 10–20% of chronically infected individuals will progress to cirrhosis and eventually develop HCC at an annual rate of approximately 2–7%. Given the remarkable efficacy of direct-acting antivirals (DAA) with cure rates over 95% of treated patients, the World Health Organization (WHO) aims for HCV elimination by 2030, which includes reducing new cases of HCV infection by 90% and decreasing the mortality rate of liver-related complications by 65% compared to 2016 [2,3,4]. However, a major challenge in HCV eradication is the complexity of HCV diagnosis, which involves a multistep process, and thus only 20% of infected individuals worldwide are currently being detected and linked to care [5]. Presently, screening for HCV infection involves two-step testing, including detecting antibodies to HCV (anti-HCV), followed by confirmation by detecting HCV RNA quantification [6,7,8]. Among treated patients, assessment of sustained virological response (SVR) requires an undetectable HCV RNA level by a sensitive assay at 12–24 weeks after completion of DAA treatment [7,8]. Nevertheless, detecting HCV RNA level as the gold standard of viremia demands expertise with high costs and time consumption, which are major barriers in the current cascade of HCV care and challenges to achieving HCV elimination [4,9].

Serum HCV core antigen (HCV-Ag), the 21-kilodalton core protein of HCV consisting of 191 amino acids, has been recently developed and demonstrates an excellent correlation with HCV RNA quantification. As a result, the assay has been endorsed as an affordable alternative biomarker for HCV diagnosis and SVR evaluation [7,8,10,11]. In addition, the strategy combining HCV-Ag and anti-HCV to diagnose HCV infection could overcome the limitations by detecting acute HCV infection during the window period, differentiating current and past infections, and being detectable in immunosuppressed individuals, including human immunodeficiency virus (HIV)-infected patients [10,12,13]. However, the performance of HCV-Ag testing could be varied depending on analytical platforms, and currently they are not yet recommended in the clinical guidelines. Therefore, additional research is necessary to validate the diagnostic role of HCV-Ag in the assessment of active infection and evaluating antiviral treatment response.

Elecsys^®^ HCV Duo immunoassay is a new electrochemiluminescence (ECLIA) immunoassay that simultaneously detects both HCV-Ag and anti-HCV in human serum and plasma. Moreover, the assay is the first commercially available test that provides the simultaneous and independent results of each biomarker [14]. A previous study demonstrated the excellent efficacy of Elecsys^®^ HCV Duo in diagnosing HCV infection in blood donors, with a high sensitivity of 99.6% and specificity of 99.94%, which enables earlier detection of active HCV infection and prevents further viral transmission [14]. In fact, the Elecsys^®^ HCV Duo immunoassay has demonstrated equal or superior diagnostic performance compared to other commercial HCV-Ag and anti-HCV tests, including the Monolisa HCV Ag-Ab ULTRA V2, Murex HCV Ag/Ab Combination, and HCV-Ag mono-test [14]. Nonetheless, its efficacy for evaluating chronic infection in independent cohorts is limited, and its role in monitoring treatment response after DAAs is absent.

Our study aimed to validate the efficacy of Elecsys^®^ HCV Duo immunoassay for the diagnosis of chronic HCV infection and the assessment of SVR post-DAA treatment in patients with or without HIV infection. With the recent need for simplified strategies to manage HCV infection, our data demonstrated that the Elecsys^®^ HCV Duo immunoassay effectively detected active HCV infection, irrespective of HIV status. This could be an appropriate and affordable option for the management of chronic HCV infection among high-risk populations, particularly in countries with resource-limited settings.

## 2. Materials and Methods

### 2.1. Study Design

The diagnostic performance of Elecsys^®^ HCV Duo immunoassay was validated using stored blood samples from 100 healthy controls and 200 patients with chronic HCV infection defined by positive anti-HCV and detectable HCV RNA for at least 6 months. The blood samples of HCV patients were collected from previous studies conducted by the Center of Excellence in Hepatitis and Liver Cancer, Faculty of Medicine, Chulalongkorn University (IRB 483/59), and the HIV-Netherlands Australia Thailand Research Collaboration (HIV-NAT) (IRB 161/45). In these patients, blood samples were collected before and 12 or 24 weeks after DAA treatment. SVR was defined by undetectable HCV RNA level (<12 IU/mL). Demographic and virological characteristics of enrolled patients, including age, sex, HCV genotype, and HCV RNA at baseline, were obtained from medical records. HCV genotypes were identified through nucleotide sequencing of the core and NS5B regions, followed by phylogenetic analysis. The lower limit and upper limit of the HCV RNA detection were <12 IU/mL and 100,000,000 IU/mL, respectively. Details of HCV genotype identification and HCV RNA quantification were described in a previous study [15].

Blood samples of healthy controls and pre- and post-treatment serum samples of the patients were tested for HCV-Ag and anti-HCV with Elecsys^®^ HCV Duo immunoassay (Roche Diagnostics International Ltd., Rotkreuz, Switzerland) according to the manufacturer’s instructions using the ECLIA method. HCV-Ag was considered positive at a coefficient of variation (COI) ≥ 1.0, while the positivity of anti-HCV was COI ≥ 1.0. HCV RNA quantification as the reference was performed by real-time quantitative reverse-transcription polymerase chain reaction (RT-PCR) (Abbott Molecular Inc. Des Plaines, IL, USA).

### 2.2. Statistical Analysis

All statistical analyses were conducted using SPSS software (version 29.01; IBM Corp., NY, USA). Categorical variables are presented as frequency (%), and continuous variables are shown as mean and standard deviation. The Elecsys^®^ HCV Duo immunoassay’s sensitivity, specificity, positive predictive value (PPV), and negative predictive value (NPV) were calculated for both HCV diagnosis and SVR assessment after DAA and are represented as percentages and at 95% confident interval (95%CI). Regarding active HCV infection at baseline, sub-results of HCV-Ag, anti-HCV, and the combination were assessed for diagnostic performance. Conversely, only HCV-Ag results were used for SVR evaluation. Diagnostic accuracy was determined by the receiver operating characteristic (ROC) curve.

This current study was reviewed and approved by the Ethics Committee and the Institutional Review Board (IRB) at the Faculty of Medicine, Chulalongkorn University, Bangkok, Thailand (IRB 149/66).

## 3. Results

### 3.1. Patients’ Characteristics

Table 1 demonstrates the demographic and virological characteristics of patients with chronic HCV infection. In brief, the majority of patients were male (86.0%), and the mean age of overall cohort was 43.09 years. Among 200 HCV-infected patients, 136 (68%) had HIV coinfection, and most of them had HCV genotype 1 of 156 (78.0%) followed by HCV genotype 3 (8.0%). Of note, 92 (46.0%) participants were treated with sofosbuvir-based regimens and 108 (54.0%) individuals were treated by other DAA regimens such as elbasvir and grazoprevir. Among them, 193 (96.5%) achieved SVR as defined by undetectable HCV RNA level at weeks 12 or 24 after stopping antiviral therapy.

### 3.2. The Performance of Elecsys^®^ HCV Duo Immunoassay for HCV Diagnosis

Table 2 demonstrates the results of Elecsys^®^ HCV Duo immunoassay before treatment (baseline) and its diagnostic performance for active HCV infection. Among HCV-infected patients, 198 (99.0%) and 175 (87.5%) had positive anti-HCV and HCV-Ag, respectively. When combined, the positivity rate of Elecsys^®^ HCV Duo immunoassay reached 99.5%.

To assess HCV diagnostic performance, 100 healthy participants were included as the negative control group. In the entire cohort, anti-HCV and HCV-Ag showed a sensitivity of 99.00% and 87.50%, respectively, with a high specificity of 100%. Combining both anti-HCV and HCV-Ag, the sensitivity and specificity for HCV diagnosis were 99.50% and 100%, respectively. As a result, the assay had a high PPV and NPV of 100% and 99%, respectively. In addition, the diagnostic accuracy of combining anti-HCV and HCV-Ag was excellent, with an area under the ROC curve (AUROC) of 0.998 (95%CI 0.992–1.00, *p* < 0.001). In subgroup analysis, the combination of anti-HCV and HCV-Ag showed similar sensitivity and specificity for diagnosing HCV infection in patients, with 100% sensitivity and 100% specificity in mono-infection and 99.3% sensitivity and 100% specificity in coinfection (Supplementary Table S1).

### 3.3. The Performance of HCV-Ag for SVR Assessment

Among 193 patients who achieved SVR, 188 (97.4%) had negative HCV-Ag. In the entire cohort, the sensitivity and specificity of HCV-Ag for SVR determination were 57.14% and 97.41% (Table 3). Additionally, the assay had a PPV and NPV of 44.44% and 98.43%, respectively. As a result, the false negative rate of detecting HCV-Ag was 1.57%. Furthermore, the diagnostic accuracy of HCV-Ag for SVR assessment exhibited an AUROC of 0.773 (95%CI 0.543–1.003, *p* = 0.020). The subgroup analyses of patients with mono-infection and coinfection are shown in Supplementary Tables S2 and S3, respectively. HCV-Ag showed an excellent diagnostic performance in HCV mono-infected patients with a sensitivity and specificity of 100%. However, the sensitivity declined in coinfected individuals to 50%, while maintaining a comparable specificity of 96.15%.

## 4. Discussion

Elecsys^®^ HCV Duo immunoassay, a promising test that simultaneously detects anti-HCV and HCV-Ag to diagnose HCV infection, remains to be validated by a real-world cohort [14]. Our study revealed that combining anti-HCV and HCV-Ag had a high sensitivity (99.5%) and specificity (100%) in detecting active HCV infection. Our findings were consistent with a previous study by Majchrazk et al., which revealed a sensitivity of 99.6% and specificity of 99.94% [14]. In addition, the diagnostic performance of Elecsys^®^ HCV Duo immunoassay in our study was comparable to other combined anti-HCV/HCV-Ag tests, such as Murex HCV Ag/Ab Combination (DiaSorin, Italy), which demonstrated a sensitivity of 100% and specificity of 95.4% [16]. Similarly, Monolisa HCV Ag-Ab ULTRA and Monolisa HCV Ag-Ab ULTRA V2 (Bio-Rad Laboratories Inc., Marnes-la-Coquette France) displayed sensitivities of approximately 92–99% and specificities of 95–100%, depending on the study population [14,17,18,19,20].

Combining all key HCV testing into a single visit in the clinics could improve the efficiency of cascade of care and enhance treatment uptake for HCV elimination. Previous data suggest the benefit of adding HCV-Ag to anti-HCV during the window period of acute infection and in subgroups of immunosuppressed populations [10,12,13,14]. In this study, we expanded its diagnostic performance into chronic HCV infection, which is a major public health problem worldwide. Of note, approximately 70% of our participants had HIV coinfection, and our results demonstrated that Elecsys^®^ HCV Duo immunoassay displayed similar diagnostic accuracy, regardless of HIV status, which was in line with the previous report [14]. Together, these data indicate that Elecsys^®^ HCV Duo immunoassay is not affected by the presence of HIV coinfection, as previously described as a main limitation when using prior generations of anti-HCV assays.

For SVR assessment, the HCV-Ag sub-result showed a good diagnostic accuracy (AUROC 0.773), with a high specificity but a relatively low sensitivity. The reduced diagnostic accuracy of HCV-Ag post-SVR compared to baseline (AUROC 0.938) might be explained by the difference in their sensitivities in detecting viremia. Moreover, the kinetics of HCV-Ag during and after DAA therapy might also contribute to the discrepancy between HCV-Ag and HCV RNA [21]. Previous studies utilizing the Architect core antigen assay by Abbott Diagnostics for SVR assessment have reported reduced accuracy during treatment but good accuracy at 12 weeks post-treatment, with a sensitivity ranging from 88.9% to 100% and excellent specificity of 97.7% to 100% [15,22,23,24].

**Figure 1:**
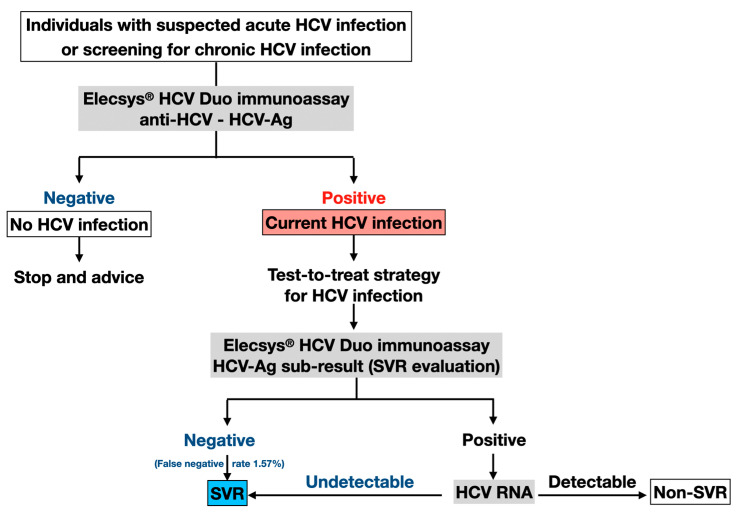
Proposed clinical application of Elecsys^®^ HCV Duo immunoassay. HCV, hepatitis C virus; anti-HCV, HCV antibodies; HCV-Ag, HCV core antigen.

According to the decreased sensitivity of HCV-Ag sub-results in SVR assessment, we propose that patients with post-treatment positive HCV-Ag should undergo additional HCV RNA testing to confirm the presence of detectable HCV RNA, as outlined in Figure 1. Nonetheless, the HCV-Ag sub-results of the Elecsys^®^ HCV Duo test demonstrated high specificity and NPV across the entire cohort, with an acceptable low false negative rate of 1.57%. In this regard, our data might indicate that HCV-Ag could be helpful for assessing SVR after DAA therapy. As more than 95% of patients achieved SVR in the DAA era, a negative HCV-Ag result could strongly indicate SVR and reduce the need for HCV RNA confirmatory tests [25].

Our report is the first study to validate the performance of the Elecsys^®^ HCV Duo immunoassays for diagnosing active HCV infection, as well as to investigate its role in assessing SVR. A strength of our study was the high proportion of patients with HIV coinfection (68%), who should theoretically benefit from adding HCV-Ag to the diagnostic test. However, our study has several limitations. Firstly, the prevalent HCV genotype in our study was genotype 1. A previous study using Elecsys^®^ HCV Duo immunoassays had patients with HCV genotypes 1–6 and showed excellent diagnostic performance [14]. Therefore, validation of the diagnostic performance of Elecsys^®^ HCV Duo immunoassays in patients infected with different genotypes is further needed. Secondly, the discordance of HCV-Ag and HCV RNA in assessing SVR might be associated with the small sample size in subgroup analysis. Upon reviewing the non-SVR patients in Supplementary Table S4, two out of three patients in a subgroup of HIV-HCV coinfection had HCV RNA below 3000 IU/mL, which could result in false negative HCV-Ag findings [26]. Thus, a larger cohort comparing HCV mono- and coinfection is warranted to confirm the performance of HCV-Ag for SVR evaluation.

Given the high prevalence of HCV infection in some populations, such as people who inject drugs, men who have sex with men, and prisoners, it is essential to distinguish active HCV infection from past exposure with viral clearance. For instance, a recent meta-analysis has demonstrated a high prevalence of anti-HCV positivity (17.7%) in prisoners worldwide, ranging between 10 and 30% over five continents [24]. Thus, simplified testing strategies are necessary among these high-risk groups to enhance treatment uptake for HCV eradication and prevent further transmission to the general population [27,28,29]. In this regard, the Elecsys^®^ HCV Duo immunoassay is a simple and affordable assay to identify individuals with chronic HCV infection as a single-step test that could lead to improved linkage to care and treatment uptake (Figure 1), particularly in resource-limited settings with high HCV prevalence and transmission potential.

## Figures and Tables

**Table 1:** Demographic and virological characteristics of patients with HCV infection.

Baseline Characteristics	Study Participants, *N* = 200 *n* (%)/Mean ± SD
Male sex	172 (86.0%)
Age (years)	43.09 ± 10.52
HIV coinfection	136 (68%)
HCV RNA before treatment Log (IU/mL)	4,762,560.73 ± 5,732,416.37 6.33 ± 0.72
HCV genotypes 1/3/4/6/unknown	156 (78.0%)/16 (8.0%)/2 (1.0%)/5 (2.5%)/21 (10.5%)
DAA regimens Sofosbuvir-based regimens Others	92 (46.0%) 108 (54.0%)
SVR	193 (96.5%)

HCV, hepatitis C virus; HIV, human immunodeficiency virus; DAA, direct-acting antiviral; *N*, number; SD, standard deviation; SVR, sustained virological response.

**Table 2:** The diagnostic performance of Elecsys^®^ HCV Duo immunoassay before treatment.

Population	anti-HCV		HCV-Ag		anti-HCV-HCV-Ag	
	Positive	Negative	Positive	Negative	Positive *	Negative
**Healthy control** (*N* = 100)	0 (0%)	100 (100%)	0 (0%)	100 (100%)	0 (0%)	100 (100%)
**HCV-infected patients** **(Entire cohort)** *N* = 200	198 (99.0%)	2 (1.0%)	175(87.5%)	25 (12.5%)	199 (99.5%)	1 (0.5%)
**HCV mono-infection** (*N* = 64)	64 (100%)	0 (0%)	56 (87.5%)	8 (12.5%)	64 (100%)	0 (0%)
**HCV-HIV coinfection** (*N* = 136)	134 (98.5%)	2 (1.5%)	119 (87.5%)	17 (12.5%)	135 (99.3%)	1 (0.7%)
**HCV diagnosis (entire cohort)**	**anti-HCV**		**HCV-Ag**		**anti-HCV-HCV-Ag**	
**Sensitivity**	99.00% (96.05–99.83%)		87.50% (81.92–91.60%)		99.50% (96.82–99.97%)	
**Specificity**	100% (95.39–100%)		100% (95.39–100%)		100% (95.39–100%)	
**Positive predictive value**	100% (97.63–100%)		100% (97.32–100%)		100% (97.64–100%)	
**Negative predictive value**	98.04% (92.41–99.66%)		80.00% (71.70–86.41%)		99.01% (93.82–99.95%)	

* Either anti-HCV or HCV-Ag positive. HCV, hepatitis C virus; anti-HCV, HCV antibodies; HCV-Ag, HCV core antigen; HIV, human immunodeficiency virus. Data are shown as *n* (%) and 95% confidence interval as appropriate.

**Table 3:** The diagnostic performance of HCV-Ag for SVR assessment.

SVR Assessment	HCV-Ag	
	Positive	Negative
**Total (*N* = 200)**	9 (4.5%)	191 (95.5%)
● **Detectable HCV RNA (non-SVR, *N* = 7)**	4	3
● **Undetectable HCV RNA (SVR, *N* = 193)**	5	188
**Sensitivity**	57.14% (20.24–88.19%)	
**Specificity**	97.41% (93.73–99.04%)	
**Positive predictive value**	44.44% (15.34–77.35%)	
**Negative predictive value**	98.43% (95.11–99.59%)	

HCV, hepatitis C virus; HCV-Ag, HCV core antigen; SVR, sustained virological response. Data are shown as *n* (%) and 95% confidence interval as appropriate.

## Data Availability

The datasets generated and/or analyzed during the current study are not publicly available due to the hospital’s safety regulations, but they are available from the corresponding author upon reasonable request.

## Supplementary Materials

The following supporting information can be downloaded at: https://www.mdpi.com/article/10.3390/diagnostics14192179/s1, Table S1: The diagnostic performance of the Elecsys^®^ HCV Duo immunoassay before treatment in the subgroups of HCV mono-infection and HCV-HIV coinfection. Table S2: The diagnostic performance of HCV-Ag after DAA treatment for SVR assessment in a subgroup of patients with HCV mono-infection. Table S3: The diagnostic performance of HCV-Ag after DAA treatment for SVR assessment in a subgroup of patients with HCV-HIV coinfection. Table S4: Details of Non-SVR patients.
