# Supplementary material for: Performance of Elecsys® HCV Duo Immunoassay for Diagnosis and Assessment of Treatment Response in HCV Patients with or without HIV Infection
Source: Diagnostics (Basel). 2024 Sep 29;14(19):2179. doi: 10.3390/diagnostics14192179 (PMC11475452; doi:10.3390/diagnostics14192179)
Supplement: Supplementary file 1 [file diagnostics-14-02179-s001.zip › diagnostics-3168056-supplementary.pdf]

Table S1. The diagnostic performance of Elecsys® HCV Duo immunoassay before treatment in the subgroups of HCV mono-infection and HCV-HIV coinfection.

HCV mono-infection	anti-HCV	HCV-Ag	anti-HCV-HCV-Ag
**Sensitivity**	100% (92.9-100%)	87.5% (76.3-94.1%)	100% (92.9-100%)
**Specificity**	100% (95.4-100%)	100% (95.4-100%)	100% (95.4-100%)
**Positive predictive value**	100% (92.9-100%)	100% (92.0-100%)	100% (92.9-100%)
**Negative predictive value**	100% (95.4-100%)	92.6% (85.5-96.5%)	100% (95.4-100%)
**HCV-HIV coinfection**	**anti-HCV**	**HCV-Ag**	**anti-HCV-HCV-Ag**
**Sensitivity**	98.5% (94.2-99.7%)	87.5% (80.5-92.3%)	99.3% (95.4-99.9%)
**Specificity**	100% (95.4-100%)	100% (95.4-100%)	100% (95.4-100%)
**Positive predictive value**	100% (96.5-100%)	100% (96.1-100%)	100% (96.6-100%)
**Negative predictive value**	98.0% (92.4-99.7%)	85.5% (77.5-91.1%)	99.0% (93.8-99.9%)

HCV, hepatitis C virus; anti-HCV, HCV antibodies; HCV-Ag, HCV core antigen; HIV, human immunodeficiency virus; DAA, direct-acting antiviral.

Table S2. The diagnostic performance of HCV-Ag after DAA treatment for SVR assessment in a subgroup of patients with HCV mono-infection

SVR Assessment	HCV-Ag	
	Positive	Negative
**Total (N = 64)**	1 (1.6%)	63 (98.4%)
• **Detectable HCV RNA (non-SVR, N = 1)**	1	0
• **Undetectable HCV RNA (SVR, N = 63)**	0	63
**Sensitivity**	100% (5.46-100%)	
**Specificity**	100% (92.84-100%)	
**Positive predictive value**	100% (5.46-100%)	
**Negative predictive value**	100% (92.94-100%	

HCV, hepatitis C virus; HCV-Ag, HCV core antigen; SVR, sustained virological response.
Data are shown as n (%) and 95% confidence interval as appropriate.

Table S3. The diagnostic performance of HCV-Ag after DAA treatment for SVR assessment in a subgroup of patients with HCV-HIV coinfection

SVR Assessment	HCV-Ag	
	Positive	Negative
**Total (N = 136)**	8 (5.9%)	128 (94.1%)

• **Detectable HCV RNA (non-SVR, N = 6)**	3	3
• **Undetectable HCV RNA (SVR, N = 130)**	5	125
**Sensitivity**	50% (13.95-86.05%)	
**Specificity**	96.15% (90.80-98.58%)	
**Positive predictive value**	37.5% (10.24-74.11%)	
**Negative predictive value**	97.66% (92.78-99.39%)	

HCV, hepatitis C virus; HCV-Ag, HCV core antigen; SVR, sustained virological response.
Data are shown as n (%) and 95% confidence interval as appropriate.

Table S4. Details of non-SVR patients

No.	HCV-Mono or HCV-HIV	HCV RNA (IU/ml) after DAA treatment	HCV RNA log	HCV-Ag	HCV-Ag COI
1	HCV-mono	658,937	5.82	Positive	1.61
2	HCV-HIV	3,619,272	6.56	Positive	26.4
3	HCV-HIV	4,460,000	6.65	Positive	7.69
4	HCV-HIV	7,905,679	6.90	Positive	2.22
5	HCV-HIV	12	1.08	Negative	0.663
6	HCV-HIV	1,808	3.26	Negative	0.591
7	HCV-HIV	2,899,246	6.46	Negative	0.582

COI, coefficient of variation; DAA, direct-acting antiviral; HCV, hepatitis C virus; HCV-Ag, HCV core antigen; HIV, human immunodeficiency virus; No., Number.
